# Investigating the coach's eye when evaluating and selecting 3 × 3 basketball players

**DOI:** 10.3389/fpsyg.2026.1756995

**Published:** 2026-03-26

**Authors:** Nora Cermak, Till Koopmann, Lena Siebert, Jörg Schorer, Karen Zentgraf

**Affiliations:** 1 Department of Movement Science and Training in Sports, Institute of Sport Science, Goethe University Frankfurt, Frankfurt am Main, Germany; 2 Sport and Movement, Institute of Sport Science, Carl von Ossietzky University Oldenburg, Oldenburg, Germany

**Keywords:** basketball, coach's eye, fast-and-frugal tree, heuristic, logistic regression, methodological comparison, multidimensional, selection

## Abstract

Although recent studies recommend making multidimensional selection decisions based on subjective and objective data, in practical settings coaches often decide subjectively. When trying to understand coaches' selection decisions, it is important to regard different theories of decision-making when analyzing predictors. Thus, this study examined predictors for selection decisions by comparing approaches of the theory of both unbounded (logistic regression) and bounded rationality (fast-and-frugal tree, FFTree). Accordingly, this study's aim was twofold: (1) investigate talent aspects in 3 × 3 basketball, and (2) compare different statistical approaches to this investigation. Regarding the underlying process of selection decisions, analyses focused on interrater agreement of subjective assessments and the relevance of different talent aspects. A total of 46 3 × 3 basketball players (female *n* = 23, age range: 15.8–18.6 years) were assessed during the final stage of the German national selection system. Data included motor performance tests, anthropometrics, and coaches' subjective assessments of technical, tactical, and psychological parameters, as well as ratings of the relevance of different talent aspects. Variables were first reduced and then analyzed using logistic regression and FFTree. Interrater reliability was assessed via intraclass correlation coefficients (ICC), and graphical analyses examined talent aspects relevance. Both logistic regression (Nagelkerke *R*^2^ = 0.70) and FFTree indicated that the subjective assessments component was the most important predictor for selection. The ICC revealed poor to moderate consistency between coaches (female players = 0.38; male players = 0.51). Graphical comparisons and descriptive statistics suggest differences between national and regional coaches in their assessments of the relevance of different talent aspects such as current performance. Although logistic regression and FFTree yielded similar main results, FFTree visualized and identified a prioritization between cues. Therefore, FFTrees are considered as an appropriate method for future studies on selection decisions, as they reflect decision-making under limited information and time pressure present in selection settings. Player selection in 3 × 3 basketball appears highly subjective, as indicated by low interrater reliability between coaches. To enhance quality and transparency, selection decisions should be made by a team of coaches based on clearer, shared criteria and be supported by actuarial approaches such as FFTrees.

## 1 Introduction

In many sports, player selection aiming to choose the most “talented,” promising, and/or appropriate players is essential due to limited resources (e.g., financial, personnel, facilities; Till and Baker, 2020; Williams and Reilly, 2000). These decisions can influence not only the player's development and career, but also the success of the respective association or institution. Despite the great importance of such decisions, they are often nontransparent processes that can be influenced by a variety of factors. These factors can relate to the athlete (e.g., anthropometrics, motor performance, motivation), but also the person responsible for the decision (e.g., experiences, subjective beliefs) or the structural factors (e.g., limited spots on the squad; Johnston and Baker, 2020). To better understand selection decisions and be able to optimize them, a crucial step is to know the most relevant predictors and understand the processes underlying this decision. Furthermore, despite increased focus on athlete selection in recent years, certain contexts and sports remain largely unexamined, including 3 × 3 basketball.

In practice, selecting players for further development opportunities or specific squads is one of the main tasks of coaches in most sports, particularly at higher levels (Xiang et al., 2024). Contrary to research recommendations, coaches often make their selection decision based on their rather subjective “gut feeling” as part of their coach's eye (Cermak et al., 2024; Den Hartigh et al., 2018; Lath et al., 2021; Roberts et al., 2019). Yet, it is not entirely clear which players' aspects are reflected most strongly in the coach's eye, and, in particular, whether coaches assess player's current performance, their future potential, or a combination of the two, even though research has found a strong link between performance potential and current performance (Barraclough et al., 2024; Gilson et al., 2025). This raises the question regarding which aspect (current performance or performance potential) is reflected more strongly in the coach's eye. Lath et al. (2021) characterized the coach's eye as intuitive, experience-based, subjective, and holistic. The latter attribute describes the idea that the coach's eye is shaped by multiple sources of information and impressions of a player, resulting in a multidimensional assessment. Which information and how one coach interprets it is believed to be influenced by the coach's experience and subjective opinion, for example, by characteristics of other players they have worked with successfully in the past. Therefore, to understand coaches' selection decisions, it is important to know what information they consider.

Previous studies have reported possible predictors for selection decisions and the information contained in the coach's eye can be both subjective and objective. Numerous studies have elucidated motor performance tests and anthropometric measurements as relevant for selection decisions as well as inheriting satisfying prognostic validity for later peak performance (e.g., Johnston et al., 2018; Schorer et al., 2017; Sliedrecht et al., 2025). Although research results across multiple sports on the role of general motor performance tests in talent selection are heterogeneous, most studies find differences in these test results between talented players and their “lower performing” peers in favor of talented players (Sliedrecht et al., 2025). In a recent review and meta-analysis, Han et al. (2023) examined which factors discriminate elite players from nonelite players of varying ages (under 12 to adult) in basketball. They found that alongside motor performance (e.g., sprint, countermovement jump), it was mainly anthropometric variables such as body height and body weight that differed significantly between elite and nonelite basketball players (Han et al., 2023).

However, the transferability of these findings to 3 × 3 basketball is limited, as not only rules, playing time, and tournament mode but also tactical and technical requirements differ substantially from 5 vs. 5 basketball (Figueira et al., 2022). Studies comparing 5 vs. 5 basketball and 3 × 3 basketball, for example, in terms of internal and external loads, shooting efficiency, and structure of shooting showed clear differences between the two sports (e.g., Erčulj et al., 2020; Willberg et al., 2023b). These differences could also affect talent selection and its predictors. The only study in 3 × 3 basketball investigated the prognostic validity for talent selection of anthropometric, motor performance, and maturation-related predictors (Schmitz et al., 2024). As in studies on 5 vs. 5 basketball, the study found that anthropometric and maturation-related parameters play a significant role in player selection for both female and male players. Moreover, motor performance variables validly predicted selection in female players but not in male players. Schmitz et al. (2024) only utilized objective measures, whereby sport-specific aspects, such as tactics and technique, are not taken into account, even though they differ between 5 vs. 5 and 3 × 3 basketball. These sport-specific variables even show higher prognostic validity than general factors when predicting selection decisions (Koopmann et al., 2020). Sport-specific tests assess more performance-related aspects, as they are representative of match demands and have higher task specificity than general motor performance tests (Sieghartsleitner et al., 2019a). To evaluate sport-specific parameters, such as technique or tactical behavior, coaches' assessments can be used (Höner et al., 2021; Koopmann et al., 2022; Leyhr et al., 2021). Due to their high expertise in their specific sport, coaches' subjective assessments offer the opportunity to assess additional important areas (e.g., technical execution of sport-specific movements, psychological attributes, etc.). These subjective assessments are often time- and cost-effective and offer an opportunity to evaluate specific aspects, as coaches base their assessments on their experiences with talented athletes (Jones et al., 2003; Zuber and Charbonnet, 2023). To improve the quality of coaches' assessments, the instrument should include a brief definition of the aspect assessed and a predefined scale, should be discussed with the coaches in advance, and the assessments should be made independently (Musculus and Lobinger, 2018). Previous research on assessments of motivation has shown that subjective coaches' assessments can be less biased than self-reports (Zuber and Charbonnet, 2023). However, it should be kept in mind that coaches' assessments are subjective and thus not unbiased. To reduce such biases, the mean assessment of multiple coaches should be used instead of a single-coach-rating (Johnston and Baker, 2020).

As shown by previous research, a prediction made by using multidimensional data, including subjective as well as objective data, shows higher prediction accuracy (Sieghartsleitner et al., 2019b). Therefore, both categories should be implemented as a basis for decision-making. To examine potential subjective and objective predictors of selection as a human decision made by coaches, any modeling of this decision-making process should also consider relevant psychological theories. As pointed out by previous research, different ways of making selection decisions may be relevant (Bar-Eli et al., 2024). Through this, more information on relevant predictors for accurately predicting selection decision could be provided. Depending on the specific circumstances, different approaches can be relevant, and combining them can therefore support coaches in their decision-making. The present article focuses on two concepts, bounded and unbounded rationality, as the two opposite ends of the decision-making spectrum. To examine predictors for talent selection, most previous studies have used a binomial logistic regression model (e.g., Cermak et al., 2024; Höner et al., 2021; Koopmann et al., 2022; Schmitz et al., 2024). This analytical approach can be attributed to the theory of unbounded rationality (Gigerenzer and Gaissmaier, 2011). This requires all information (subjective and objective data, e.g., body height, sprint times, technique, competitiveness) to be available and implemented in the decision-making process in order to achieve an optimal decision. Accordingly, in logistic regression, a binary variable (e.g., selected vs. not selected) is explained by including all available information (e.g., subjective and objective predictors). In contrast, the theory of bounded rationality assumes that only a limited amount of information and time are available for decision-making, and that a satisficing decision is therefore made with a certain degree of uncertainty (Simon, 1979). Fast-and-frugal trees (FFTrees) are one way of analyzing selection decisions according to the latter theory. FFTrees classify objects into two categories (e.g., selected vs. not selected) by searching through cues (e.g., different predictors) that are sorted according to their relevance (highest to lowest probability for a correct prediction) in a determined order (Green and Mehr, 1997; Marewski et al., 2010). FFTrees prioritize limited cues and as soon as a decision is made based on a cue reaching a specific cut-off value, all lower-level cues are ignored (Martignon et al., 2008). For example, in an FFTree consisting of a first cue (e.g., grip strength) and a second cue (e.g., flexibility), a fictional player A with high grip strength values would be classified as selected without considering flexibility. Whereas for fictional player B with low grip strength, the next cue in the example, flexibility, would be considered for classification. FFTrees have already been applied in the field of competitive sport (Siebert et al., 2025), because they have been shown to be robust against overfitting, making them suitable for small samples (Gigerenzer and Brighton, 2009; Luan et al., 2011; Martignon et al., 2008). However, FFTrees have not been used in the context of player selection, although they can be as accurate as logistic regressions or even outperform them (Phillips et al., 2017). Considering the potential of FFTrees to advance talent research through reflecting human decision-making under time pressure and with limited information, it is important to examine their applicability in selection decisions (Bar-Eli et al., 2024). Given that coaches' decisions are unlikely to be made with all available information taken into account, bounded rationality is considered a more applicable approach. Following both theories, the present study is an opportunity to examine key differences in their application and adequacy. Examining both theories in terms of their methodological requirements and results makes it possible to have comparable results with previous research as well as to compare both approaches. From the knowledge of the decision (outcome) and possible information considered (predictors), both approaches aim to reconstruct the decision-making process.

As well as examining relevant factors for selection decisions, it is important to understand the processes underlying these decisions, and therefore to better understand the coach's eye and the factors influencing it. The subjective nature of the coach's eye makes it particularly relevant to examine the extent to which coaches provide consistent subjective evaluations. Earlier studies have found low levels of agreement among coaches when assessing players (Bergkamp et al., 2022; Fitzgerald et al., 2025; Roberts et al., 2020). To the best of the authors' knowledge, no studies have yet used intraclass correlation coefficients (ICC) to examine the interrater reliability of coaches' subjective assessments of players' performance potential in the context of player selection. These could be used to investigate how consistent different coaches are in their subjective assessments. In sports which rely on subjective judgments (e.g., figure skating, ski jumping), previous research has shown high interrater reliability between different judges (Braeunig, 2025). These judgment are complex and need to be made under time pressure, but in contrast to coaches' assessments, the judgments are based on clear criteria, with the weighting of individual aspects clearly defined (Braeunig, 2025). Variability in the coach's eye and therefore prediction accuracy may result, at least in part, from differences in the perceived importance of specific aspects, because, unlike judges, coaches usually do not follow predefined criteria when forming their assessment (Peringa et al., 2025; Roberts et al., 2019). These specific aspects remain relatively consistent across different coaches, although the value or weight coaches assign to each aspect may differ (Roberts et al., 2019). However, the extent to which different weightings of talent aspects influence the coach's eye is rarely examined in talent research (Bergkamp et al., 2022; Peringa et al., 2025). To describe and understand how coaches combine information to develop their coach's eye and to compare it with actuarial alternatives (e.g., FFTrees), previous research has used, for example, the Lens Model (Peringa et al., 2025). The Lens Model serves as a conceptual framework describing how individuals make a judgment about an outcome that is yet unknown by combining information (cues) that are probabilistically related to the outcome (Brunswik, 1952; Peringa et al., 2025). Its name comes from the schematic representation of the model, in which reality is perceived and interpreted through a “lens” consisting of different cues.

In summary, the aim of this article is twofold:

I) Gain a deeper understanding of player selection, coaches' decision-making, and talent aspects in 3 × 3 basketball. Therefore, the first aim addresses the following research questions: (Ia) Is the selection decision based primarily on performance potential or on current performance? It was hypothesized that, even if the aim is to select the most promising players, the coach's eye would be closely associated with current performance, since it is visible directly and current performance would correlate strongly with performance potential. (Ib) Which variables can predict player selection in 3 × 3 basketball? It was hypothesized that 3 × 3-specific assessments would be the most important predictors for player selection in adolescent 3 × 3 basketball followed by anthropometrics and motor performance, as this has also been shown in other sports. (Ic) How consistent are different coaches in their subjective assessments? A low level of interrater reliability was expected since there are often no established criteria to assess different aspects and because of biases within coaches' assessments and its high subjectivity and variability, as shown by Roberts et al. (2020). (Id) How do coaches weigh different aspects of talent in 3 × 3 basketball?
II) The second aim of this article is to compare two different theories of decision-making and their respective statistical approaches for investigating predictors of player selection. This raises the following research question: (IIa) How do predictors of logistic regression and FFTrees differ from one another? It was assumed that the most important predictor in both analyses would be the same, but that FFTrees uses less variables for a similar prediction. This may be based on the fact that bounded rationality assumes limited amount of information and time for a satisficing decision.

## 2 Methods

**Figure 1:**
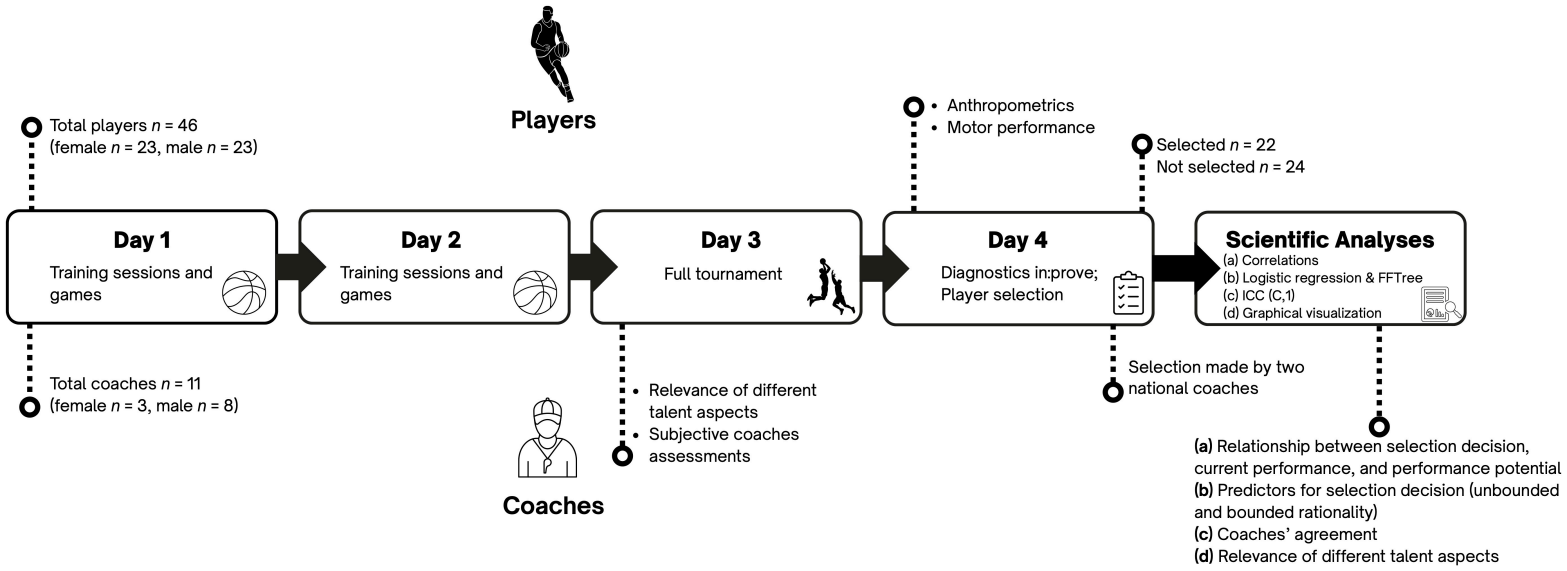
Procedure of selection camp, data collection, and scientific analyses.

Data collection took place during the second, and final, selection stage for the national under-17 and under-18 German 3 × 3 basketball teams. The national selection camp lasted 4 days (January 3–6, 2025) and took place in Bielefeld, Germany. On the first 2 days, both training sessions and games were conducted. The third day consisted of a full tournament, and on the fourth day, diagnostics were conducted as part of the in:prove project (funded by the German Federal Institute of Sport Science [Bundesinstitut für Sportwissenschaft, BISp], reference number: 081901/21-28). The detailed procedure for the selection camp and data collection is described below and shown in Figure 1. The study was approved by the Ethics Committee of the Faculty of Psychology and Sports Sciences of Goethe University Frankfurt and by the Ethics Committee of the Faculty of Medicine of Justus Liebig University Giessen. Both were in accordance with the Declaration of Helsinki for human research. Informed consent was obtained from all participants and parents or legal representative(s) of participating athletes included in the study.

### 2.1 Participants

A total of 46 players (age: *M* = 16.9, *SD* = 0.65 years) participated in the selection camp (female *n* = 23, male *n* = 23). All players were preselected at the first selection stage by the national coaches to participate in the second camp (September 2024 in Bad Hersfeld, Germany).

Coaches' assessments were collected from 11 coaches (age: *M* = 33.2, *SD* = 8.0 years; female *n* = 3, male *n* = 8). These 11 coaches included two national coaches, who made the selection decision, and nine regional coaches who supported the national coaches during the selection camp but were not involved in the decision-making. All coaches were licensed basketball coaches with either a German A- (highest license in Germany; *n* = 5) or a C-license (lowest license; *n* = 6). The coaching experience was between 5 and 27 years with a mean of 11.5 years (*SD* = 6.5 years).

### 2.2 Data collection

#### 2.2.1 Coaches' selection decisions

On the last day, the two national coaches decided on the selections for the national squad that were then communicated to each player in private conversations. After the camp, each player's selection status was collected for this study (*selected n* = 22, *not selected n* = 24).

#### 2.2.2 Subjective coaches' assessments

On the third morning of the camp, coaches were provided with tablets (iPad, 9th generation, Apple Inc., Cupertino, USA) to assess players using a digital observation sheet. This sheet included an overview of all players and a specific evaluation sheet for each player. The evaluation sheet contained the player's name, a picture to facilitate identification, and the aspects to be evaluated (example can be found in the Supplementary Datasheet 1). Each coach was asked to assess seven different aspects for each player: *current performance, performance potential*, and five sport-specific aspects (*technique 1-point throw, technique 2-point throw, tactical understanding and decision-making, competitiveness and will to win*, and *willingness to play a team role*). The first two scales, which assessed *current performance* and *performance potential*, were adapted from Büsch et al. (2021). The sport-specific aspects were selected based on current literature on relevant talent aspects in basketball (Koopmann et al., 2025a,b; Larkin et al., 2023; Schmitz et al., 2024). Coaches were asked to rate each player on each aspect on a scale from 1 (*very low*) to 10 (*very high*) in relation to all attending players of the same sex. A 10-point Likert scale was chosen because it had been proven suitable for coaches in the authors' previous studies, and the data could be treated as interval scaled for the statistical procedures (Wu and Leung, 2017). The observation sheet was explained to all coaches, and all questions were clarified in advance. Coaches had the possibility to consider all impressions from the current and previous days, including games and training sessions, as well as general observations during the rest of the day. Additionally, coaches had the option not to assess a specific aspect of a player or the entire player if they felt unable to provide a fair assessment. As this was the second selection stage and regional coaches are responsible for the regional teams and for the preliminary selection, all coaches were already familiar with at least some of the players. All assessments could be altered at will until the final submission. Coaches were told not to share their assessment with other coaches before submission. Two coaches assessed only one sex from the outset (one coach only female and one coach only male players) as they believed that their experience only allowed them to evaluate the respective sex. Thus, nine coaches evaluated both sexes (including the two national coaches).

#### 2.2.3 Anthropometrics

Anthropometric measures consisted of *body height* (cm), *body weight* (kg), and *wingspan* (cm). All measures were taken without shoes and to the nearest 0.1 cm using a measuring tape (Busduga, Leutkirchen, Germany) or to the nearest 0.1 kg using a digital scale (Bohmann, Hamburg, Germany). For the measurement of *body height*, the player stood upright against a wall with his/her heels together. The distance between the ground and the highest point of the skull was measured. *Wingspan* was measured as the distance between the two tips of the middle fingers in a prone position with the arms stretched out at shoulder level.

#### 2.2.4 Motor performance

All players warmed up individually before motor performance measurements. Measurements included the following tests: *sprint* (s), *countermovement jump* (cm), *drop jump* (m/s), *vertical jump* (cm), *change of direction* (s), and *chest pass* (m). Each test was carried out at least twice. If the difference between the two trial values was greater than 10%, a third trial was carried out. The best trial was used for analyses unless this differed more than 10% from the second-best trial, in which case the second-best trial was used.

*Sprinting* times for 10 meters were measured using light barriers (Witty Gate, Microgate, Bolzano, Italy; measurement accuracy = 0.4 ms) to an accuracy of 0.01 s. Players started 1 m behind the first light barriers and started independently without a formal start signal.

The *countermovement jump* was measured using a photocell system (OptoGait, Microgate, Bologna, Italy). Players were instructed to start in an upright, shoulder-width stance, then descend to a self-selected depth with an approximate knee angle of 90°. They were asked to jump as high as possible in one continuous movement. Arms were held at the hips during the entire jump.

The *drop jump* was also measured using a photocell system (OptoGait, Microgate, Bologna, Italy) to assess the Reactive Strength Index (RSI; jump height/contact time). Players dropped from a 24 cm high box and jumped as high as possible with a minimal ground contact time. Arms were held at the hips during the entire jump, as in the *countermovement jump*. A drop height of 24 cm was chosen based on previous research results recommending lower heights between 20 and 40 cm for youth athletes (Bassa et al., 2012; Birat et al., 2020), and to reduce risk of injury during our diagnostics for the elite athletes.

For the *vertical jump*, a self-built jump-and-reach apparatus similar to a Vertec was used. A 3 m-radius was marked around the apparatus from which the players had to start. Players were instructed to jump as high as possible in a jump as close to the game as possible. The maximum height of the lowest untouched plate was measured using an electro-optical rangefinder (Bosch, Gerlingen, Germany).

For the *change of direction* test, light barriers were used (Witty Gate, Microgate, Bolzano, Italy; measurement accuracy = 0.4 ms) in a test setting and protocol based on previous research (Willberg et al., 2023a). The corners and the center of a square with a side length of 5 m were marked. Cones were placed at each mark, except for the lower left corner, where the light barriers were positioned (1.5 m apart). Athletes started 1 m behind the light barriers and had to keep their hips and face facing forward at all times, which meant that some sections had to be run sideways or backward. They had to run in the following order and touch cones with the specified hand: upper left cone (left hand), center cone (right hand), upper right cone (right hand), lower right cone, (right hand), center cone (left hand), and then pass through the light barrier to stop the time (test setup can be found in the Supplementary Datasheet 2).

The *chest pass* was performed using an official 3 × 3 basketball (Wilson Sporting Goods, Chicago, United States of America). Players stood in an upright position with their feet parallel and threw the basketball as far as possible with both hands. The maximum throwing distance was measured as the distance between the starting position and the point at which the ball first touched the ground using a measuring tape (Stanley, Idstein, Germany; measurement accuracy = 0.1 cm). The ball was held in three different starting positions: center at chest height, and to the left and right at chest height. During the throw, the starting position could not be left, otherwise the trial had to be repeated.

#### 2.2.5 Relevance of different talent aspects

Subjective ratings of 12 different talent aspects for 3 × 3 basketball were gathered to evaluate how relevant coaches found these aspects. Coaches were asked to assess each aspect's relevance on a scale from 0 (*very low relevance*) to 100 (*very high relevance*) in order to ensure sufficient gradations. The coaches were not familiar with this specific scale before. However, all questions were answered in advance. The following aspects were selected based on previous research: *performance potential, motor performance, anthropometr*y, *athleticism, competitiveness, playing ability, environmental factors, tactics, technique 1-point throw, technique 2-point throw, current performance*, and *biological maturity* (form can be found in the Supplementary Datasheet 3; Koopmann et al., 2025a,b; Larkin et al., 2023; Schmitz et al., 2024).

### 2.3 Data analysis

First, point-biserial correlations were calculated separately for each sex for selection decision and *current performance*, and selection decision and *performance potential*, and a Pearson correlation for *performance potential* and *current performance*. Thereby, the assumption that the selection decision was based primarily on the *current performance*, and that *current performance* would correlate with *performance potential* was tested (Research Question Ia).

All following statistical analyses were conducted for female and male players combined. Unless otherwise indicated, missing values were replaced by the arithmetic mean of the respective sex for the respective variable. For the analyses, all data were *z*-standardized depending on sex. The means of all available coaches' assessments (except the national coaches, because they make the selection decisions) were calculated separately by sex for each of the five specific aspects assessed.

**Figure 2:**
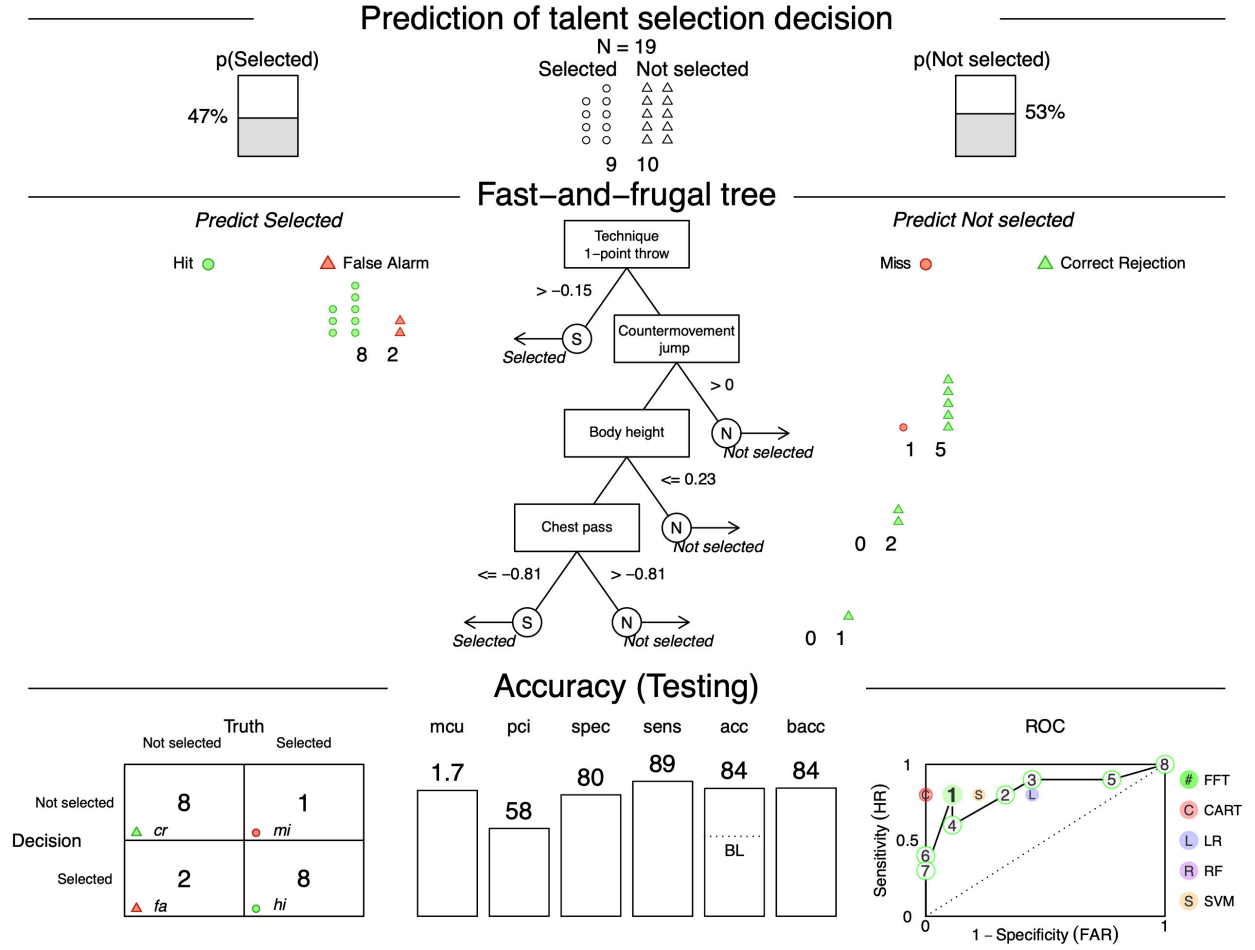
Fast-and-frugal tree for selection decision including predictive performance. Fast-and-frugal tree (FFTree) to determine the selection decision for 3 × 3 basketball players based on four cues including classification and predictive accuracy of test data from 45% of the sample calculated with the ifan algorithm and the *z* scores; acc, accuracy; bacc, balanced accuracy; CART, classification and regression trees; LR, logistic regression; mcu, mean cues used; pci, percentage cues ignored; RF, random forest; ROC, receiver operating characteristic [presenting the performance of all FFTrees from the “fan” based on the bacc with false alarm rate (FAR) on the *x* and sensitivity on the *y* axis]; sens, sensitivity; spec, specificity; SVM, support vector machine.

**Figure 3:**
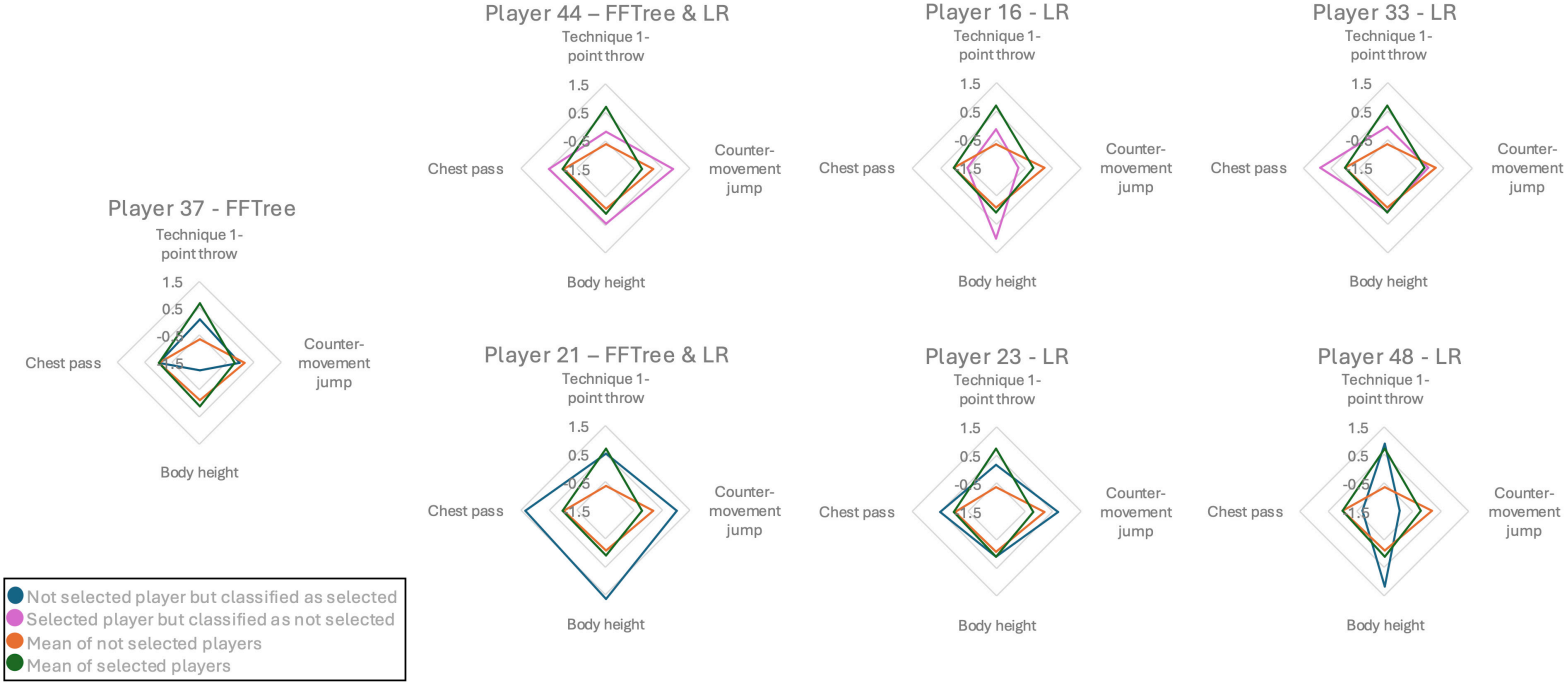
Spider charts of all misclassified players.

**Figure 4:**
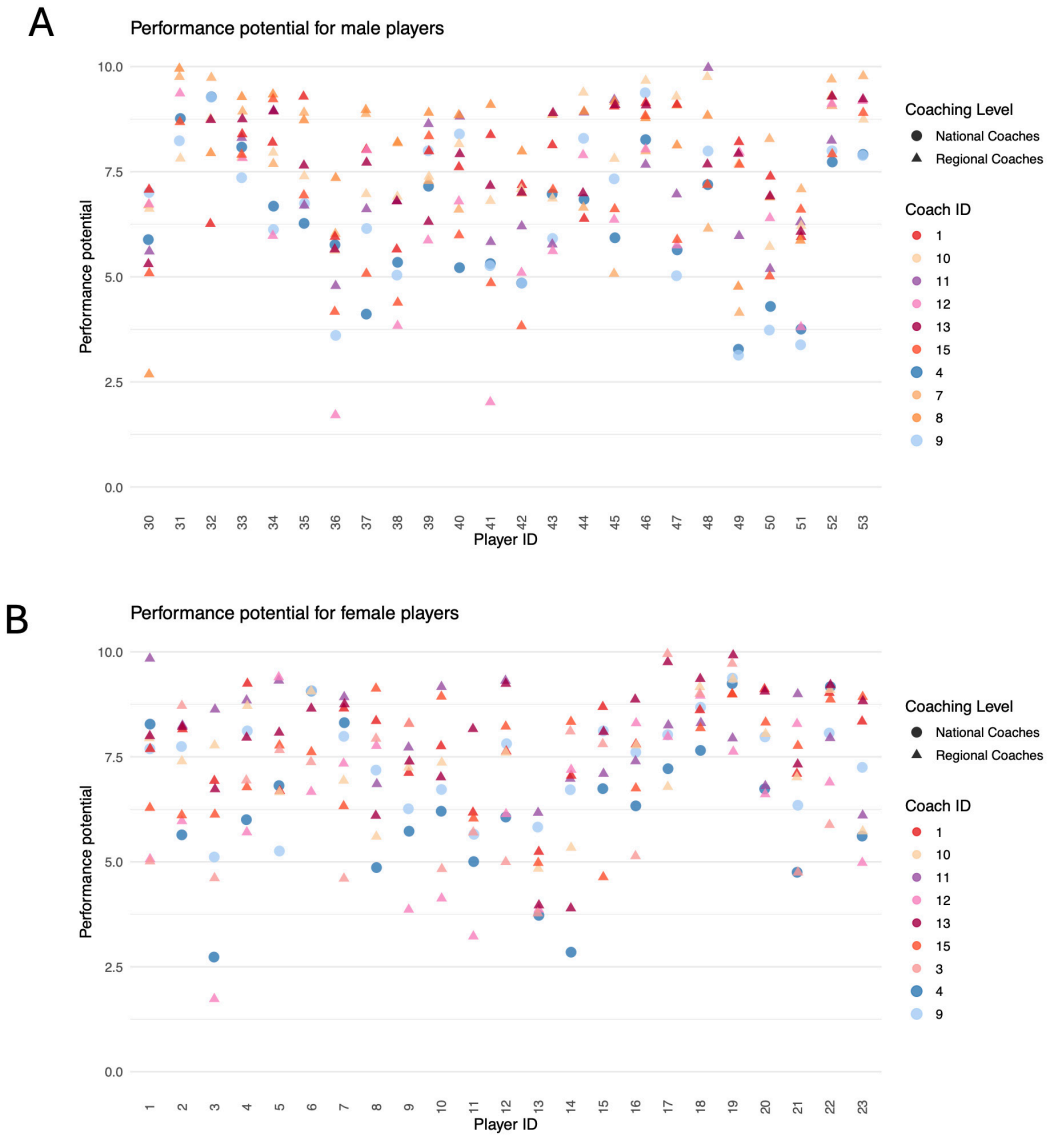
Assessed performance potential for male **(A)** and female players **(B)** (national coaches are shown as dots and regional coaches as triangles).

The correlations, principal components analysis (PCA), and binomial logistic regression were conducted using IBM SPSS Statistics 29.0.2.0 (IBM Corp., Armonk, New York, USA). The FFTrees were conducted and created using the package FFTrees 2.0.0 (Phillips et al., 2017), and for the ICC, the package psych 2.5.6 (Revelle, 2025). Both packages were used in R 4.4.2. (R Core Team, R Foundation for Statistical Computing, Vienna, Austria) and RStudio version 2024.09.1+394 (Posit team, Posit Software, PBC, Boston, MA, USA). Figures 2–4 were also created within RStudio version 2024.09.1+394 using ggplot2 package (Wickham, 2016). The statistical significance level for all tests was set at α = 0.05.

To address Research Question Ib (which subjective and objective data predict player selection best), player-related variables were reduced to prevent overfitting and multicollinearity. Variable reduction was guided by theoretically meaningful relationships and examined using a PCA (varimax rotation). One variable displayed low anti-image correlations, indicating limited shared variance with the remaining variables. Accordingly, this variable was excluded from the PCA but retained for subsequent predictive analyses as the lack of shared variance with all other variables does not preclude its inclusion but rather supports its role as an additional independent predictor. In addition, variables with the highest component loading were selected as representative variables for their respective component to facilitate interpretation and replicability of the results.

To determine the most relevant predictors for player selection and understand the decision-making process, two separate data-driven analyses were conducted and their results were compared. In both analyses, selection status (*selected* vs. *not selected*) served as the dependent respectively outcome variable, while the variables resulting from variable reduction were used as independent variables or cues. The first method was a logistic regression. All assumptions (linearity, no outliers, sphericity, sampling adequacy) were checked and fulfilled. Variables were tested for multicollinearity using Spearman correlation coefficients and using the variance inflation factor (VIF). Second, FFTrees were used following the methodological procedure indicated by previous research (Siebert et al., 2025). More specifically, representative cues were first derived from the PCA components, followed by data partitioning to assess overfitting and predictive accuracy. For the FFTrees, 55% of the data were used to train the FFTree. The training data were given slightly lower preference compared to partitioning in Siebert et al. (2025), because the sample sizes of *selected* vs. *not selected* cohorts were more balanced. The remaining 45% of the data were used to test the predictive performance of the created FFTree. Players with missing data on a given cue were excluded when analyzing that cue. Consequently, no missing data was imputed as the sample size remained unchanged even without imputation. To ensure reliable and accurate measurements, FFTrees were evaluated through repeated random partitioning (20 random FFTrees). The FFTree with the highest accuracy was selected and its results presented. The following information was used to compare the two different statistical approaches (logistic regression vs. FFTree; Research Question IIa): most important predictor, number of relevant predictors in total, percentage of correct classified players.

For in-depth analyses, spider charts were created post hoc for the few incorrectly classified players of the FFTree and the logistic regression to better understand why these cases were misclassified.

To examine whether subjective assessments of different coaches were consistent (Research Question Ic), the interrater reliability was assessed using a two-way mixed-effects model, single-rating, assessing consistency (ICC(C,1); McGraw and Wong, 1996). ICC(C,1) was calculated separately for female and male players, because two coaches assessed only one sex.

An exploratory approach using graphical visualization and descriptive statistics (*M* and *SD*) was employed to examine Research Question Id on how coaches assess the relevance of different aspects of talent in 3 × 3 basketball. Therefore, boxplots for the ratings from all regional coaches across all 12 talent aspects were visualized. Additionally, the ratings of the two national coaches were included as individual data points to make differences in coaching expertise visible.

## 3 Results

Results are presented in the following order of the research questions, arranged according to their content: Ia, Ib, IIa, Ic, and Id. To gain further insights into players, population-descriptive statistics can be found in the Supplementary Table S1.

### 3.1 Relationship between selection decision, current performance, and performance potential

The hypotheses that *current performance* would be closely related to the selection decision (*selected* vs. *not selected*) and that *current performance*, and *performance potential* would correlate were checked using point-biserial and Pearson correlations. For female players, selection decision and *current performance* correlated with *r* = 0.74, and for male players with *r* = 0.89 (see Table 1). *Current performance* and *performance potential* correlated for female players with *r* = 0.75, and for male players with *r* = 0.78 (see Table 1).

**Table 1:** Point-biserial and Pearson correlations between selection decision, current performance, and performance potential.

**Sex**	**Variable**	**Selection (1 = *selected*; 0 = *not selected*)**	***Current performance***	***Performance potential***
Female athletes	Selection *(*1 = *selected;* 0 = *not selected)*	1.00^*^	–	–
	*Current performance*	0.74^*^	1.00^*^	–
	*Performance potential*	0.82^*^	0.75^*^	1.00^*^
Male athletes	Selection *(*1 = *selected;* 0 = *not selected)*	1.00^*^	–	–
	*Current performance*	0.89^*^	1.00^*^	–
	*Performance potential*	0.62^*^	0.78^*^	1.00^*^

^*^p < 0.05.

### 3.2 Analysis of relevant selection criteria

#### 3.2.1 Variable reduction

A PCA was conducted on 13 player-related variables with varimax rotation. All variables were chosen based on previous research (Koopmann et al., 2025a,b; Larkin et al., 2023; Schmitz et al., 2024). The following variables were included: five specific subjective aspects assessed by the coaches (means of all but national coaches), *drop jump, countermovement jump, vertical jump, 10 m sprint, change of direction, body height, body weight*, and *wingspan*. Sampling adequacy was verified using the Kaiser-Meyer-Olkin test that showed a value of 0.726 (*middling* according to Kaiser and Rice, 1974). Three components with an eigenvalue greater than 1.0 were revealed and supported by the scree plot, explaining 78.8% of variance. All five specific subjective assessments (*technique 1-point throw, technique 2-point throw, tactical understanding and decision-making, competitiveness and will to win, willingness to play a team role*) correlated with component one (i.e., subjective assessments component). Variables *vertical jump, body height, body weight*, and *wingspan* correlated with component two (i.e., anthropometric component). *Drop jump, countermovement jump, 10 m sprint*, and *change of direction* correlated with component three (i.e., lower-body performance component). The variables with the highest component loadings for each component were *technique 1-point throw, body height*, and *countermovement jump*. All component loadings (loadings greater than 0.50 in bold) are shown in Table 2.

**Table 2:** Rotated component matrix for player-related variables.

Rotated component matrix			
	Component		
	1	2	3
*Technique 1-point throw*	**0.95**	0.01	0.02
*Technique 2-point throw*	**0.78**	−0.27	0.06
*Tactical understanding and decision-making*	**0.94**	−0.09	−0.08
*Competitiveness and will to win*	**0.92**	−0.14	0.08
*Willingness to play a team role*	**0.85**	−0.04	0.00
*Drop jump*	0.06	−0.03	**−0.74**
*Countermovement jump*	−0.16	0.18	**−0.85**
*10 m sprint*	0.34	0.30	**0.62**
*Change of direction*	−0.33	0.24	**0.69**
*Vertical jump*	−0.24	**0.82**	−0.32
*Body height*	−0.03	**0.89**	0.15
*Body weight*	−0.00	**0.68**	−0.49
*Wingspan*	−0.19	**0.92**	0.10

Loadings greater than 0.50 are in bold.

#### 3.2.2 Predictors for selection decision (unbounded rationality)

The logistic regression used the three variables with the highest component loading from the PCA (*technique 1-point throw, body height, countermovement jump*) and *chest pass* to predict the selection decision (*selected* vs. *not selected*). All requirements were fulfilled (VIF < 10; no outliers; linearity). The model was statistically significant, χ^2^(4) = 34.25, *p* < 0.001, and explained 70.1% (Nagelkerke *R*^2^) of the variance in selection decision, correctly classifying 84.8% of players. The only statistically significant variable was *technique 1-point throw* (*p* < 0.001, see Table 3).

**Table 3:** Binomial logistic regression.

**Predictor**	**B**	***SE* B**	**Wald**	***df***	***p***	***e^*B*^*(odds ratio)**	**95% CI**
*Countermovement jump*	−0.41	0.53	0.61	1	0.436	0.66	(0.24, 1.86)
*Chest pass*	−0.04	0.51	0.01	1	0.942	0.96	(0.35, 2.64)
*Body height*	−0.12	0.54	0.05	1	0.832	0.89	(0.31, 2.59)
*Technique 1-point throw*	3.59	1.08	10.96	1	< 0.001	36.20	(4.32, 303.09)

#### 3.2.3 Predictors for selection decision (bounded rationality)

The selected FFTree was tested on 19 players (as seen in Figure 2), of whom nine were *selected* (shown as circles) and 10 were *not selected* (shown as triangles). The selection decision was determined by all four cues (*technique 1-point throw, countermovement jump, body height*, and *chest pass*), with an average of 1.7 cues (mean cues used; mcu) required for the decision and 58% of the cues being ignored (pci). The predictive performance of the FFTree showed a specificity of 80% and a sensitivity of 89%, resulting in a balanced accuracy of 84%. The confusion matrix at the bottom left shows that from the nine *selected* players, the FFTree correctly classified eight players as *selected* (hit; hi) and one player wrongly (miss; mi). From the 10 *not selected* players, the FFTree classified eight players correctly (correct rejection; cr) and two players wrongly (false alarm; fa). Illustrating the decision process, the threshold suggests that players with an assessed *z*-standardized *technique 1-point throw* higher than −0.15 can be classified as *selected*, and all subsequent cues can be ignored. The *technique 1-point throw* as the first cue was able to correctly classify 42% of selection decisions. Players who fall below this threshold will be assessed using the following cue: their *countermovement jump* performance. This suggests that players with a *z* value higher than zero are classified as *not selected*, and those with a lower *z* value than zero will be assessed by the next cue: their *body height*. A *z*-standardized *body height* of 0.23 or lower is assessed as *not selected*. Players with a higher *z*-standardized *body height* are classified by the last cue: their *chest pass* performance. Players with a *z*-standardized *chest pass* value of −0.81 or lower are classified as *selected*, and higher as *not selected*. The receiver-operating characteristic (ROC) curve at the bottom right visualizes the sensitivity and specificity of the FFTree and other common statistical analysis methods such as logistic regression, classification and regression trees, random forest, and support vector machine.

#### 3.2.4 Comparison of logistic regression and FFTrees

Both analyses found the subjective assessments component to be the most relevant predictor for selection decision. The logistic regression included all four variables in the model and correctly classified 84.4% of the players, whereas the FFTree used an average of 1.4 cues for a decision and correctly classified 84% of the players.

#### 3.2.5 Misclassified players

To gain a clearer understanding of which players were misclassified by the two analytical approaches presented above (logistic regression and FFTrees), post hoc spider charts were generated for all misclassified players. As shown in Figure 3, two players were misclassified by both analytical approaches, one only by the FFTree, and four only by the logistic regression. For the FFTree, only players misclassified in the test dataset were included, resulting in a smaller sample and therefore a lower number of misclassified players.

Players who were classified as *not selected* but were actually *selected* showed a lower *technique 1-point throw* than the average of *selected* players, but displayed well above-average values in at least one of the other areas. In contrast, players who were classified as *selected* but belonged to the group of *not selected* showed a noticeably higher *technique 1-point throw* than the average of *not selected* players and were around the mean in most of the other aspects. Player 21, however, showed a different pattern with very high and mostly above-average values across all four variables.

### 3.3 Coaches' agreement

The hypothesis of a low interrater agreement regarding the subjective coaches' assessments was tested using ICC (C,1). ICC for the subjective assessments of *performance potential* included nine coaches for the female players (one coach needed to be excluded due to too many missing values) and ten coaches for the male players. Missing values were omitted pairwise for all ICC. The ICC (C,1) for female players showed a poor reliability (0.38, see Table 4) and a moderate reliability for male players (0.51, see Table 4).

**Table 4:** Results of ICC (C,1) for female and male players' performance potential.

**Gender**	**Intraclass correlation**	**95% CI**	***F***-test with true value 0			
			Value	*df*1	*df*2	*p*
Female	0.38	(0.24, 0.58)	6.6	22	176	< 0.001
Male	0.51	(0.36, 0.69)	11.4	22	198	< 0.001

Figure 4 presents the individual assessments for each player from all available coaches separately for female and male players. National coaches are indicated by blue dots, whereas regional coaches are represented by differently colored triangles. As can be seen for both sexes, national coaches tended, on average, to rate players lower than regional coaches. The degree of variation among coaches depended on the specific player. For instance, for Player 18, coaches exhibited a high level of agreement in their *performance potential*, whereas for Player 21, substantial discrepancies could be observed.

### 3.4 Relevance of different talent aspects

**Figure 5:**
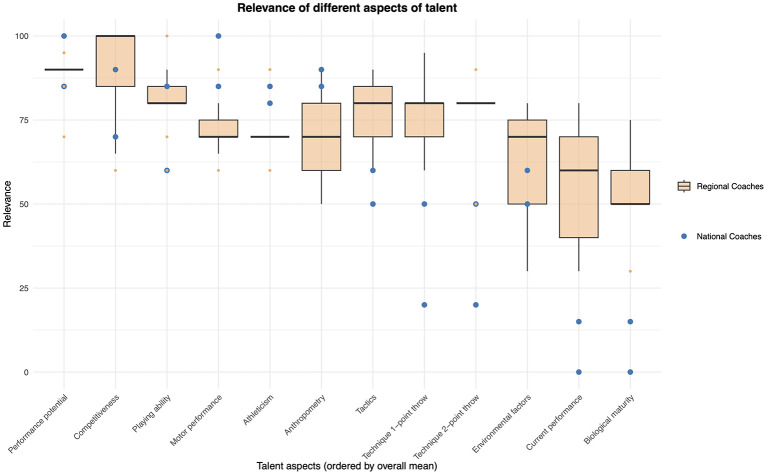
Coaches' rating of the relevance of different talent aspects.

To account for potential differences between coaches as well as the two coaching roles (national and regional coaches), individual relevance ratings of each coach for the 12 talent aspects are illustrated in Figure 5. Regional coaches are illustrated as boxplots, whereas the national coaches are shown as blue dots. Descriptive statistics are shown in Table 5. Graphical visualization and descriptive statistics suggest that, on average, coaches rated *performance potential* as the most important talent aspect (*M* = 89.1, *SD* = 7.7), whereas *biological maturity* (*M* = 45.9, *SD* = 22.3) seems to be considered the least important in 3 × 3 coaches. For most talent aspects, the relevance assigned by national and regional coaches appears similar. However, the greatest visible discrepancies were observed in *current performance* (national coaches: *M* = 7.5, *SD* = 10.6; regional coaches: *M* = 57.8, *SD* = 18.6), *technique 1-point throw* (national coaches: *M* = 35.0, *SD* = 21.2; regional coaches: *M* = 74.4, *SD* = 14.2), and *biological maturity* (national coaches: *M* = 7.5, *SD* = 10.6; regional coaches: *M* = 54.4, *SD* = 12.6), with national coaches appearing to assign lower relevance to these aspects than regional coaches.

**Table 5:** Descriptive statistics of the relevance of different talent aspects.

**Variable**	Regional coaches		National coaches		Total	
	*M*	*SD*	*M*	*SD*	*M*	*SD*
Playing ability	81.1	11.4	72.5	17.7	79.5	12.1
Anthropometry	70.6	12.9	87.5	3.5	73.6	13.4
Environmental factors	60.6	18.4	55.0	7.1	59.5	16.8
Athleticism	73.3	10.0	82.5	3.5	75.0	9.7
Motor performance	72.2	8.7	92.5	10.6	75.9	11.8
Current performance	57.8	18.6	7.5	10.6	48.6	26.5
Biological maturity	54.4	12.6	7.5	10.6	45.9	22.3
Tactics	75.6	11.0	55.0	7.1	71.8	13.1
Technique 2-point throw	74.4	14.2	35.0	21.2	67.3	21.5
Technique 1-point throw	77.2	10.9	35.0	21.2	69.5	20.8
Competitiveness	88.9	16.0	80.0	14.1	87.3	15.4
Performance potential	88.3	7.5	92.5	10.6	89.1	7.7

## 4 Discussion

The aims of the article were twofold. First aim was to gain a deeper understanding of player selection in 3 × 3 basketball, coaches' decision-making and the relevance of different talent aspects (Research Questions Ia, Ib, Ic, and Id). Second aim was to compare two analytical methods for examining selection decisions (Research Question IIa). Due to the contextual connections between Research Questions Ib and IIa, the discussion also corresponds to the order of the results (Ia, Ib, IIa, Ic, and Id).

### 4.1 Performance potential vs. current performance as the primary basis for selection decisions

Regarding Research Question Ia, the hypothesis that *current performance* and *performance potential* correlate with each other is confirmed for both sexes and is in line with previous research results (Barraclough et al., 2024; Gilson et al., 2025). Even though *current performance* and *performance potential* show a strong link, high *current performance* in youth does not necessarily translate into high performance at senior level (Barreiros et al., 2014; Güllich et al., 2023). Therefore, high *current performance* does not necessarily imply high *performance potential* (Güllich et al., 2023). The preliminary hypothesis that the selection decision is related to players' *current performance* is partially confirmed. Male players show a higher correlation between selection decision and *current performance* than between selection decision and *performance potential*, whereas for female players the opposite is the case. For female players, point-biserial correlations show a higher correlation between selection decision and *performance potentia*l. These results imply that a higher *performance potential* is more related to being *selected* for female players, and a higher *current performance* rating for male players. One reason to partly explain this difference could be that female players often have less and not year-round strength and conditioning training compared to male players (Reynolds et al., 2012). As a result, *current performance* of female players may be less meaningful due to not fully established or well-conditioned strength, which can also affect other aspects as playing style or technique. For male players, *current performance* may already be more representative of their *performance potential* and therefore more relevant for coaches' selection decisions, as it is easier to assess. Another potential and plausible explanation for these differences may be that the two national coaches have a different understanding of talent for female compared to male players. As shown by the research by (Johnston et al. 2023), blurry terminologies for ‘talent' as well as misconceptions of the differences between current performance and future potential are evident in both research and practice (Barraclough et al., 2025). Assessing *performance potential* primarily based on *current performance* without using objective or other valid measures may lead to inferior selections. However, it should be noted that both *current performance* and selection, as well as *performance potential* and selection, show high correlations, particularly among female athletes, as did *current performance* and *performance potential*. These differences should therefore not be overinterpreted and further studies are necessary.

### 4.2 Predicting player selection in 3 × 3 basketball: a comparison of bounded and unbounded rationality

To examine the most relevant subjective and objective predictors for player selection in 3 × 3 basketball (Research Question Ib), the initial 13 variables were reduced to four variables that were then used for further analyses. Regarding the PCA, all subjective aspects loaded onto a single component, indicating highly correlated predictors. This could be due to the halo effect, which assumes that a positively rated characteristic (e.g., *body height*) can positively influence the assessment of other characteristics (e.g., technique; Dennis, 2007; Thorndike, 1920). It could also be that, due to their holistic coach's eye, coaches' assessments of individual aspects are strongly influenced by their overall impression and are therefore highly interrelated.

It was hypothesized that 3 × 3-specific assessments would be the most important predictors for player selection in adolescent 3 × 3 basketball. Examining predictors for selection decision using logistic regression shows that only *technique 1-point throw*, as the representative variable for the subjective sport-specific assessments' component, is statistically significant. The subjective assessments component predicts selection status in 84.8% of the players correctly. Neither the anthropometric component, lower-body performance component, nor *chest pass* (representative for upper-body strength) emerges as statistically significant in the logistic regression, which therefore does not correspond to the hypothesis. These results are thus not in line with the findings of Schmitz et al. (2024) who found motor performance to be relevant in player selection for female but not for male players. An important difference between the current study and that of Schmitz et al. (2024) is that the latter took place at the first national selection stage. The current study, however, includes only players participating in the second and final stage at which national coaches have already preselected approximately half of the players from the first stage. Schmitz, Fleddermann and Zentgraf's (2024) speculation that there could be motor-performance benchmarks that must be reached for lower selection stages would also fit our data, because female players at the second national selection stage show higher homogeneity in most tests than in Schmitz et al. (2024). These can also be decisive in the previous stages of selection as a benchmark that needs to be fulfilled to get to the second and last selection stage. At the last stage, these motor performance aspects may no longer discriminate between *selected* and *not selected* players.

In line with the results of the logistic regression, the final FFTree also shows that the subjective assessments component is the most important cue that correctly classifies eight out of nine *selected* players. According to the hypothesis, all four variables are included as cues in the FFTree, but they do not appear in the expected order. Reasons for this could be the same as those suspected for the results of the logistic regression, because even though all four variables are used as cues, only a mean of 1.7 cues is necessary for the classification, underlying the frugal characteristic of the FFTree. Simon (1955) conceptualized that decision makers use a relatively small number of cues for complex problems, employing the less-is-more effect. More ecologically, decisions are characterized by uncertainties in which the decision process is structured by a noncompensatory search of information, and the decision process is characterized more by a striving for a satisfactory solution rather than by maximizing (Gigerenzer and Goldstein, 1996). FFTrees are therefore well-suited for predicting selection decisions, because they break down the decision process into the most relevant cues and are easy to understand and interpret due to their transparent structure (Luan and Reb, 2017). The second cue (*countermovement jump*) and the fourth cue (*chest pass*) both appear to have counterintuitive thresholds, as a higher *countermovement jump* height and a higher *chest pass* distance indicate non-selection. It should be noted, however, that players with an above average *countermovement jump* are only classified as *not selected* when their first cue (*technique 1-point throw*) is below average. One possible explanation for this finding is the high level of homogeneity in the motor performance variables. Consequently, at the second and final selection stage investigated in the present study, motor performance may not sufficiently predict selection decisions. As suggested by the graphical visualization of all misclassified players, Player 44, who was classified as *not selected* after the second cue showed an above-average *countermovement jump* height but received a below-average assessment in *technique 1-point throw*.

In practice, decision makers can identify the most important predictors for the selection decision by the cue order, and use this for future selection decisions. Moreover, the thresholds can give insights into reference values appropriate for a *selected* 3 × 3 basketball player. As seen in Figure 2, an exemplary basketball player that receives a high evaluation on the *technique 1-point throw* (subjective assessments component) will be *selected* directly without any need to check for any other predictors in more detail. Even though FFTrees greatly reduce the coach's selection decision and thus the coach's eye to single cues, FFTrees offer the possibility of mapping potential implicit prioritization of information that flows into the holistic coach's eye. It should also be noted that individual variables were selected for the analysis with the intention of representing their respective component. Although *technique 1-point throw* is identified as the most relevant cue, this indicated that sport-specific aspects are generally very important for talent selection decisions, since *technique 1-point throw* was the representative variable for the subjective assessments component. To be able to use FFTrees and, in particular, the given thresholds in practice, it would be necessary to calculate the FFTree using a significantly larger sample size and thus be able to include more information. Studies have shown that the larger the sample size used to calculate the FFTree, the more stable its outcome is (Phillips et al., 2017).

Regarding the comparison of logistic regression and FFTree (Research Question IIa), both analyses found the subjective assessments component to best predict the selection outcome. FFTrees visualize heuristic decisions in a selection decision while using only limited information, in contrast to logistic regressions (Luan et al., 2011). This is also evident in the number of variables used for the prediction. While logistic regression includes all variables in the model, FFTree uses an average of only 1.4 cues for a decision. When comparing the results of logistic regression and FFTree, it should be noted that the FFTree is constructed with a training dataset and evaluated on a testing dataset, adhering to one core heuristic principle defined as “how well models of heuristics predict new data” (Marewski et al., 2010, p. 82; Raab, 2012). Logistic regression, in contrast, seeks to achieve the best possible model fit for the entire dataset, thus having a higher risk for overfitting. Even though the logistic regression showed a slightly higher accuracy of 84.8% than the FFTree (84%), these numbers are not directly comparable, as for logistic regression the accuracy only describes how good the model fits the current data but does not consider a possible risk of overfitting. One way to compare the accuracy of the FFTree with that of other statistical approaches is through the ROC. In the FFTree approach, the risk of overfitting is tested through data partitioning following the principles of heuristics formulated by Marewski et al. (2010) and Raab (2012). A comparison of statistical approaches on predicting new data through a testing dataset can be seen from the ROC curve (Figure 2, right corner). The relation of sensitivity to specificity is illustrated by the ROC curve with optimal values located in the upper left corner. The ROC shows that the FFTree can reach nearly similar or even exceed predictive performance compared to alternative classification methods such as logistic regression. The results and especially the comparison of the two methods indicate that FFTrees are a suitable statistical method for examining predictors for player selection, as they model selection decisions under limited information and time pressure.

When examining the misclassified players in both the logistic regression and the FFTree using spider charts, it becomes evident that most misclassifications are due to the subjective assessments component. Based on the previous results, it seems reasonable to assume that the subjective assessments of the regional coaches differ considerably from those of the national coaches. This would explain why some players were not selected despite receiving high scores, particularly in the subjective assessment. To better understand this specific selection decision, as well as the general process behind the selection decisions, examining the agreement between different coaches could provide further insights. One player, however, presents a different picture: Despite very high scores and above-average scores in almost all areas, this player is *not selected*, although both analyses classify the player as *selected*. Another possible reason why this player was *not selected* may be that coaches have made an incorrect decision by not selecting this player. There may be various reasons for this, including structural factors such as maximum number of places in the squad. A future analysis of this player's performance development in relation to other players of this sample, could provide interesting points of observation.

### 4.3 Inter-coach consistency in subjective assessments

Analysis of the interrater reliability (Research Question Ic) confirms the assumption of low intercoach agreement and reliability and is consistent with the results of earlier studies (Roberts et al., 2020). The ICC for female players shows poor and for male players moderate reliability (Koo and Li, 2016). The selection decision could therefore be strongly influenced by the individual coach who is responsible. As defined by Lath et al. (2021), the coach's eye is based on the coach's experience in various ways such as working with and developing different player types. Another explanation could be the different experience levels of the coaches in the rather young sport and system of 3 × 3 basketball. The two national coaches had an average of 18.5 years of coaching experience, and both have an A-license. In comparison, the regional coaches have an average of 10.0 years of experience and mostly have a C-license. The finding that both national coaches give the players lower assessments on average could be due to their experience as national coaches or the decision-making responsibility inherent to their position. Another reason could be that coaches were already familiar with some of the players before the two selection camps. As a result, their evaluation may have been influenced not only by players' performances during the selection camp but also by previous information and impressions gained from, for example, training sessions or games. There can be many other reasons for cognitive biases within the decision-making process such as the availability heuristic, confirmation bias, or the halo effect (Mann et al., 2025). Subjective assessments may bias selection decisions, because such assessments can vary considerably not only between different coaches but also within the same coach at different times (Kite et al., 2025; Mann et al., 2025; Roberts et al., 2025). The interrater reliability among competition judges in sports is typically much higher than in the context of coaches' assessments. This could be explained by the presence of clear criteria for evaluating performance and the fact that the weighting of individual criteria is defined explicitly (Braeunig, 2025). One way to deal with biases of subjective assessments could be to incorporate machine-based approaches or so-called actuarial approaches such as FFTrees, pattern recognition analysis or other, as previous research showed that they can outperform coaches' global assessments by systematically combining variables and their weights (Barraclough et al., 2025; Den Hartigh et al., 2018; Johnston and Baker, 2020). Another way to reduce these biases could be to involve multiple coaches in the selection process, because this could bring benefits and reduce errors (Johnston and Baker, 2020).

### 4.4 Coaches' weighting of different talent aspects in 3 × 3 basketball

The graphical representation and descriptive statistics of the relevance of different talent aspects (Research Question Id) suggests that, although both groups of coaches (national and regional) appear to be relatively similar in most aspects, there seem to be certain differences between the two groups. Having a high *performance potential* is rated as the most important talent aspect which is partly in line with the results of the first analysis. Particularly notable is the very low estimated relevance of the *current performance* for the national coaches compared to the assessments of the regional coaches. This appears to be one of the major discrepancies between national coaches and regional coaches. And even though national coaches rated the relevance of *current performance* as relatively low, the results of the first analysis show that *current performance* and *performance potential* correlate highly, and that *current performance* and selection decision correlate more strongly than *performance potential* and selection decision for male players. This supports the intuitive nature of the coach's eye (Lath et al., 2021). Often criticized in the field of talent selection is that coaches select players with an above-average *current performance* but low *performance potential*, because coaches are also evaluated based on current competition results. One particular aspect of the examined sample is that, due to the regulations of the International Basketball Federation, no international competitions will take place for the selected squads in the next year or two, meaning that coaches do not face pressure when making their selection.

Overall, the present findings contribute to a better understanding of player selection in a less-well-studied sport and provide a foundation for further research. Our results highlight the subjectivity of player selection in 3 × 3 basketball, indicating that decisions are strongly influenced by the individual coach. As noted, the selection decision can have a major impact on an athlete's career path, affecting which resources and training opportunities they receive. From a broader perspective, this underscores the need for more transparent and structured selection processes that combine objective performance variables with subjective assessments, as previous research has already shown (Höner et al., 2021; Sieghartsleitner et al., 2019b).

## 5 Limitations

One limitation of this study is the sample size that required a reduction of variables for analyses. However, this study was performed in a real-life selection setting within a sport with a small team size and at a high performance and selection level. Even though variables were reduced using a PCA to retain as much information as possible, the reduction of variables prior to the logistic regression and FFTree, and the resulting selection of only the variable with the highest loading (besides *chest pass*), may lead to information loss or the omission of potential predictors. A PCA was performed because variable reduction was necessary and the resulting components were interpretable in terms of content. Only variables with the highest loading were used for our analyses. Even though they must not be the strongest predictors, due to the high correlation of variables loading onto one component, no big difference was assumed regarding the results of the analyses. Though the reduction of variables was necessary due to the prerequisites of the analyses and the comparability of both methods, this was accompanied by further limitations. The theory of unbounded rationality assumes that all information is available and is included in the decision-making process. However, due to the reduction of variables and the setting of a selection camp (where not all kinds of information can be collected due to limited resources) not ‘all' information was available and therefore could not be included in the analysis. Nevertheless, both analyses attempted to include as much variation of the available information as possible. Further, the FFTree used for comparison was calculated using a data partitioning approach, where the logistic regression used the entire dataset. Therefore, the accuracy of the logistic regression is not directly comparable with the predictive accuracy of the FFTree. For a fair comparison, the dataset used for the logistic regression would also have to be split into training and testing subsets. This was not done in the present study, in line with previous studies. However, the ROC curve can instead be used to evaluate and compare the predictive accuracy of the FFTree. For the analysis, the 10-point Likert scale was assumed to be interval scaled. Future studies could include even more levels. It should also be noted that, due to the situation and their position, coaches were already familiar with at least some of the players. This was not considered in the analysis, as it applied to almost all coaches and, consequently, to most players to some extent. Another limitation is that all motor performance tests were general tests and not sport-specific. And although studies in 5 vs. 5 basketball have already shown that general motor performance and anthropometric tests are important (Han et al., 2023), their prognostic validity for long-term talent prediction is questionable due to previously mentioned influences such as training experience (Lidor et al., 2009). The only sport-specific data were the subjective variables. Future studies could examine additional sport-specific or game-related variables such as game statistics (e.g., rebounds and shooting accuracy). Further self-reports could be used in addition to coaches' assessments to gain explanatory power. With regard to the last research question, no inferential statistical analysis was possible due to the small group sizes (national coaches *n* = 2, regional coaches *n* = 9).

## 6 Conclusion and practical implications

The subjective assessments component showed to be the most important predictor for player selection in 3 × 3 basketball in both the logistic regression and FFTree analysis. Even though anthropometric and motor performance data was included in both analyses, they seem to hardly differentiate between selected and not selected players at this final selection stage. Regarding the comparison of both analytic approaches in predicting selection outcomes, FFTrees used less information achieving a comparable accuracy of correctly classified players by applying a training and test dataset. Given their ability to model selection decisions under limited information and time pressure, FFTrees offer a promising analytical approach for investigating selection decisions in future research.

However, subjective assessments show low reliability across different coaches, indicating a high degree of subjectivity. It appears to be important for coaches in the same system to work toward a mutual understanding of how they assess players (i.e., evaluation) and what they look for and use for their decisions (i.e., selection). For future player selections, multiple coaches should be involved because this can reduce the influence of a single subjective assessment, thereby ensuring a more comprehensive and balanced selection process. Additionally, actuarial approaches such as FFTrees could be used to support selection decisions. Greater agreement among coaches could also be achieved through a uniform understanding of relevant talent aspects and their individual weighting. And although the coach's eye is holistic and already integrates multidimensional data, establishing clearer and shared criteria for selection decisions could make the process more transparent and therefore more reliable and valid.

## Data Availability

The datasets presented in this article are not readily available because of sensitivity and recognizability of the data of elite athletes. However, a subset of the data, provided as *z* score means with standard deviations and fully de-identified, can be obtained on request from the corresponding author. Requests to access the datasets should be directed to cermak@sport.uni-frankfurt.de.
