# Supplementary material for: Investigating the coach's eye when evaluating and selecting 3 × 3 basketball players
Source: Front Psychol. 2026 Mar 26;17:1756995. doi: 10.3389/fpsyg.2026.1756995 (PMC13063375; doi:10.3389/fpsyg.2026.1756995)
Supplement: Supplementary file 1 [file Table_1.pdf]

TABLE S1

Descriptive statistics of the players (female and male as total and separated in selected and not-selected) as mean and standard deviation (SD)

		Female Players			Male Players		
		Selected	*Not selected*	Total	*Selected*	*Not selected*	Total
		Mean ± *SD*	Mean ± *SD*	Mean ± *SD*	Mean ± *SD*	Mean ± *SD*	Mean ± *SD*
objective	*Chest pass* (m)	10.6 ± 1.3	10.3 ± 0.9	10.4 ± 1.1	14.4 ± 2.1	14.9 ± 1.6	14.7 ± 1.8
	Vertical jump (cm)	282.7 ± 9.1	283.1 ± 8.7	282.9 ± 8.7	322.3 ± 8.2	328.7 ± 9.9	326.1 ± 9.6
	Countermovement jump (cm)	28.3 ± 5	29.4 ± 2.9	28.8 ± 4.1	37.0 ± 6.0	41 ± 6.2	39.2 ± 6.3
	Drop jump (m/s)	1.2 ± 0.2	1.2 ± 0.4	1.2 ± 0.3	1.5 ± 0.4	1.5 ± 0.2	1.5 ± 0.3
	10m sprint (s)	2.0 ± 0.1	2.0 ± 0.1	2.0 ± 0.1	1.8 ± 0.1	1.8 ± 0.1	1.8 ± 0.1
	Change of direction (s)	7.8 ± 0.4	7.9 ± 0.3	7.8 ± 0.4	7.0 ± 0.6	7.2 ± 0.5	7.1 ± 0.5
	Body weight (kg)	72.0 ± 8.4	67.2 ± 6.6	69.6 ± 7.8	83.7 ± 14.9	83.9 ± 9.5	83.8 ± 11.8
	Body height (cm)	176.1 ± 4.6	175.2 ± 4.2	175.7 ± 4.3	190.4 ± 7.9	189.1 ± 6.8	189.7 ± 7.1
	Wingspan (cm)	179.4 ± 5.9	178.9 ± 6.7	179.2 ± 6.1	199 ± 8.3	195.1 ± 7.6	196.8 ± 8
	Age of peak height velocity (years)	12.8 ± 0.4	12.6 ± 0.2	12.7 ± 0.3	13.5 ± 0.7	13.5 ± 0.8	13.5 ± 0.7
subjective	*Technique 1-point throw* (scale 1-10)	7.9 ± 0.7	6.4 ± 1.1	7.2 ± 1.2	7.8 ± 0.6	6.2 ± 0.8	6.9 ± 1
	Technique 2-point throw (scale 1-10)	7.3 ± 1.0	5.7 ± 1.1	6.5 ± 1.3	7.6 ± 1.0	6.5 ± 0.7	7.0 ± 1.0
	Tactical understanding and decision-making behavior (scale 1–10)	8.0 ± 0.9	6.2 ± 1.3	7.1 ± 1.4	7.6 ± 0.7	6.3 ± 1.1	6.9 ± 1.1
	Will to win and competitiveness (scale 1-10)	8.5 ± 0.8	6.9 ± 0.9	7.7 ± 1.2	8.4 ± 0.8	6.9 ± 0.7	7.5 ± 1.1
	Team role (scale 1–10)	8.3 ± 0.7	7.2 ± 0.9	7.8 ± 1.0	8.0 ± 0.8	7.2 ± 1.1	7.5 ± 1.1

Note. Subjectively assessed variables were rated on a scale from 1 (*very low*) to 10 (*very high*).
