# Supplementary material for: Investigating the coach's eye when evaluating and selecting 3 × 3 basketball players
Source: Front Psychol. 2026 Mar 26;17:1756995. doi: 10.3389/fpsyg.2026.1756995 (PMC13063375; doi:10.3389/fpsyg.2026.1756995)
Supplement: Supplementary file 2 [file Data_Sheet_1.pdf]

NAME

General aspects

Current performance

Assess NAME's current overall performance level in comparison to all male/female players present.

	1	2	3	4	5	6	7	8	9	10	
very low	☐	☐	☐	☐	☐	☐	☐	☐	☐	☐	very high

Performance potential

Assess NAME's potential for future performance improvement up to his/her peak performance age in comparison to all male/female players present.

	1	2	3	4	5	6	7	8	9	10	
very low	☐	☐	☐	☐	☐	☐	☐	☐	☐	☐	very high

Sport-specific aspects

Technique 1-point throw

Assess NAME's number of variations in finishing techniques at or on the drive to the basket in comparison to all female/male players present.

	1	2	3	4	5	6	7	8	9	10	
very low	☐	☐	☐	☐	☐	☐	☐	☐	☐	☐	very high

Technique 2-point throw

Assess the quality of NAME's shooting technique on 2-point distance shots in comparison to all female/male players present.

	1	2	3	4	5	6	7	8	9	10	
very low	☐	☐	☐	☐	☐	☐	☐	☐	☐	☐	very high

Tactical understanding and decision-making

Assess NAME's tactical game understanding and decision-making in comparison to all female/male players present.

	1	2	3	4	5	6	7	8	9	10	
very low	☐	☐	☐	☐	☐	☐	☐	☐	☐	☐	very high

Competitiveness and will to win

Assess NAME's training commitment and willingness to perform in comparison to all female/male players present.

	1	2	3	4	5	6	7	8	9	10	
very low	☐	☐	☐	☐	☐	☐	☐	☐	☐	☐	very high

Willingness to take on a role within the team

Assess NAME's ability to assume a specific role within the team in comparison to all female/male players present.

	1	2	3	4	5	6	7	8	9	10	
very low	☐	☐	☐	☐	☐	☐	☐	☐	☐	☐	very high

Carl von Ossietzky Universität Oldenburg

in:prove LEISTUNGSRESERVE INDIVIDUALISIERUNG

Deutscher Basketball Bund DBB

GOETHE UNIVERSITÄT FRANKFURT AM MAIN
