# Supplementary figures and images for: Investigating the coach's eye when evaluating and selecting 3 × 3 basketball players
Source: Front Psychol. 2026 Mar 26;17:1756995. doi: 10.3389/fpsyg.2026.1756995 (PMC13063375; doi:10.3389/fpsyg.2026.1756995)

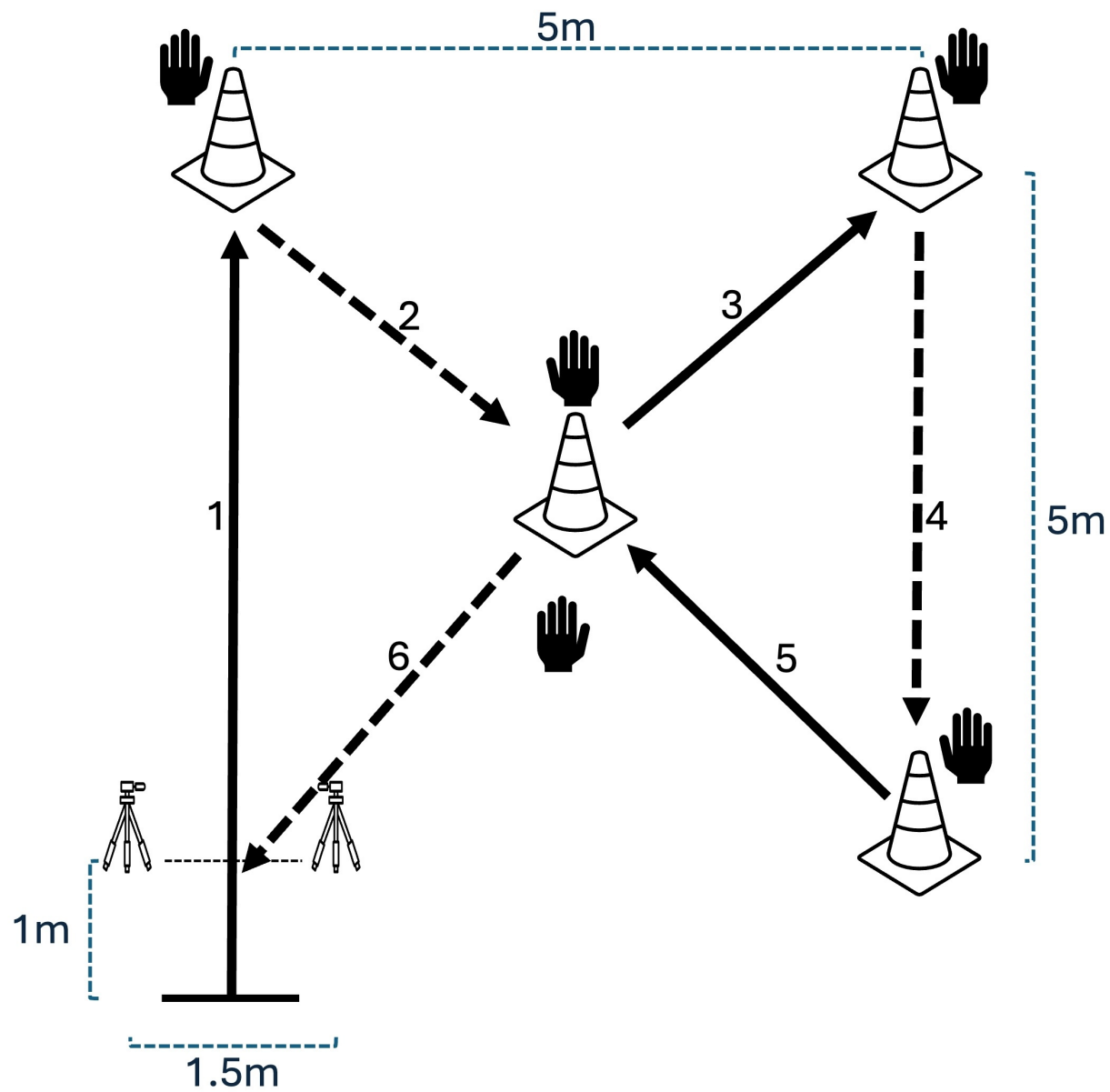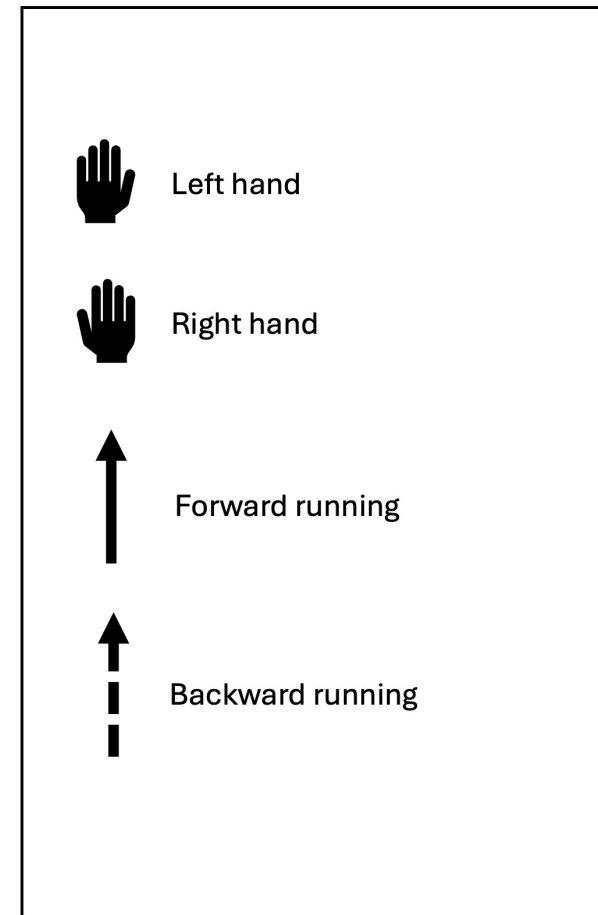

Supplement: Supplementary file 3 [file Data_Sheet_2.pdf]
