# Supplementary material for: Investigating the coach's eye when evaluating and selecting 3 × 3 basketball players
Source: Front Psychol. 2026 Mar 26;17:1756995. doi: 10.3389/fpsyg.2026.1756995 (PMC13063375; doi:10.3389/fpsyg.2026.1756995)
Supplement: Supplementary file 4 [file Data_Sheet_3.pdf]

Relevance of different talent aspects

Please assess the relevance of different talent aspects on a scale from 0 (very low relevance) to 100 (very high relevance) and enter the corresponding values in the fields on the left.

Relevanz	Talent aspect
	Playing ability
	Anthropometry
	Environmental factors
	Athleticism
	Motor performance
	Current performance
	Biological maturity
	Tactics
	Technique 1-point throw
	Technique 2-point throw
	Competitiveness
	Performance potential

Carl von Ossietzky Universität Oldenburg

in:prove LEISTUNGSRESERVE INDIVIDUALISIERUNG

Deutscher Basketball Bund DBB

GOETHE UNIVERSITÄT FRANKFURT AM MAIN
